# COLORECTAL CANCER SCREENING (CRC) DISPARITIES: A ZIP CODE–LEVEL ANALYSIS

**DOI:** 10.1093/geroni/igac059.1859

**Published:** 2022-12-20

**Authors:** Roshni Singh, Carla Parraga, Rachel Lin, Leonardo Tamariz, Ana Palacio

**Affiliations:** University of Miami Miller School of Medicine, Miami, Florida, United States; University of Miami Miller School of Medicine, Miami, Florida, United States; University of Miami Miller School of Medicine, Miami, Florida, United States; University of Miami Miller School of Medicine, Miami, Florida, United States; University of Miami Miller School of Medicine, Miami, Florida, United States

## Abstract

CRC is the third leading cause of cancer-related deaths among older adults in the US. CRC screening can prevent disease by early identification, yet there are disparities in CRC screening. This study aimed to determine the impact of race, social determinants, and geographic location at zip-codes level on CRC screening.We conducted a retrospective cross-sectional study of CRC screening among different races, evaluating the relationship with the social deprivation index (SDI) and annual income as health determinant factors using the public available data of 2016-2019 CDC 500 cities project and PLACES project 2020 database combined with 2019 American Community Survey for zip code-based analysis. We conducted a multivariate analysis and a confirmatory factor analysis among race, income, lack of health insurance, access to check-up visits and SDI.Increasing SDI tertile increased the likelihood of being Black and Hispanic and having lower median household income (p< 0.01). Lack of health insurance and lower regular checkup visits were less common in the third tertile of SDI (p< 0.01). The multivariate analysis showed that being black, Hispanic, having a lower income, not having health insurance, not having regular check-ups and SDI were related to decreased screening. In the confirmatory factor analysis, the variables most associated with decreased screening are SDI and access to health insurance.Race, SDI, insurance status, socioeconomic status, all impact CRC screenings, but the two most important factors are SDI and access to healthcare. These data may help implement interventions that specifically target these barriers to promote CRC screenings within disadvantaged communities.

